# Contribution of glycogen in supporting axon conduction in the peripheral and central nervous systems: the role of lactate

**DOI:** 10.3389/fnins.2014.00378

**Published:** 2014-11-25

**Authors:** Tom W. Chambers, Timothy P. Daly, Adam Hockley, Angus M. Brown

**Affiliations:** ^1^School of Life Sciences, Queen's Medical Centre, University of NottinghamNottingham, UK; ^2^Department of Neurology, University of WashingtonSeattle, WA, USA

**Keywords:** glycogen, white matter, axon, lactate, biosensor

## Abstract

The role of glycogen in the central nervous system is intimately linked with the glycolytic pathway. Glycogen is synthesized from glucose, the primary substrate for glycolysis, and degraded to glucose-6-phosphate. The metabolic cost of shunting glucose via glycogen exceeds that of simple phosphorylation of glucose to glucose-6-phosphate by hexokinase; thus, there must be a metabolic advantage in utilizing this shunt pathway. The dogmatic view of glycogen as a storage depot persists, based on initial descriptions of glycogen supporting neural function in the face of aglycemia. The variable latency to conduction failure, dependent upon tissue glycogen levels, provided convincing evidence of the role played by glycogen in supporting neural function. Glycogen is located predominantly in astrocytes in the central nervous system, thus for glycogen to benefit neural elements, intercellular metabolic communication must exist in the form of astrocyte to neuron substrate transfer. Experimental evidence supports a model where glycogen is metabolized to lactate in astrocytes, with cellular expression of monocarboxylate transporters and enzymes appropriately located for lactate shuttling between astrocytes and neural elements, where lactate acts as a substrate for oxidative metabolism. Biosensor recordings have demonstrated a significant steady concentration of lactate present on the periphery of both central white matter and peripheral nerve under unstimulated baseline conditions, indicating continuous cellular efflux of lactate to the interstitium. The existence of this lactate pool argues we must reexamine the “on demand” shuttling of lactate between cellular elements, and suggests continuous lactate efflux surplus to immediate neural requirements.

## Introduction

In the last 20 years interest in brain energy metabolism, and in particular specific cellular substrate utilization and trafficking of metabolites between neural elements and glia has witnessed a rapid growth, which shows no sign of abating. This is due to several factors which may be identified as follows: the realization that certain neurological conditions e.g., Alzheimer's disease, may in part be due to metabolic disturbance in the brain (Cai et al., 2012), improved or novel technologies revealing previously unavailable information regarding metabolism (Brown et al., 2012; Barros et al., 2013), and an increased awareness of the importance of the role of lactate (Dienel and Hertz, 2001; Dienel, 2004). It is this final topic that is the subject of this review article. The dogmatic view of lactate is as a waste product, the result of incomplete metabolism of glucose, where glucose is glycolytically converted to pyruvate / lactate but not oxidatively metabolized (Stryer, 1995). The role of lactate in the periphery must not be confused with its role in the CNS. In the periphery, and in particular muscle, lactate is the result of incomplete oxidative metabolism of glucose (Stryer, 1995), and has entered the common vernacular in relation to sport e.g., lactate threshold (Messonnier et al., 2013). The lactate accumulation is due to the bodies' inability to take in sufficient oxygen during intense exercise to completely metabolize available glucose, with the shortfall between oxygen uptake and glucose availability being manifest as an accumulation of lactate. As an aside, it has recently been shown that the lactate generated in the periphery as a result of maximal exercise is not a waste product after all, and crosses the blood brain barrier where it is oxidatively metabolized by the brain (Dalsgaard, 2006), although glucose is still the main energy substrate. The relationship shows a degree of metabolic co-operation between the muscles and the brain, whereby the muscle takes up all available glucose, the only substrate muscle can efficiently metabolize, for instantaneous energy production, and the brain extracts lactate from the systemic circulation, which can exceed 5 mmol l^−1^ during maximal exercise (Dalsgaard, 2006). Brain energy metabolism is more complex than that which occurs in muscle due to the variety of different cell types in the brain, each of which has individual requirements dependent upon function. Given the absolute requirements of glucose and oxygen as the main energy support of the brain, and the extremely rapid (6–8 s) loss of consciousness that occurs when blood supply to the brain is interrupted for even the shortest period of time (Rossen et al., 1943), glucose and oxygen are clearly vital commodities to the brain. Thus, it is surprising that not all glucose is oxidatively metabolized in the brain. If such were the case the respiratory quotient (RQ; the ratio of oxygen molecules consumed per glucose molecule metabolized) for the brain would be 6 (as 6 oxygen molecules are required in the metabolism of one glucose molecule, which contains 6 carbon molecules); the RQ for resting brain is in fact nearer 5.5 indicating a significant proportion of the glucose is not oxidatively metabolized. In addition this ratio decreases with increased activity, falling to values as low as 3 (Dienel, 2009). This provokes several questions: what is the benefit to the brain of incomplete glucose oxidation? Is there cellular compartmentalization with regard to glucose metabolism, and if so how does this change during increased tissue energy demand? What role does glycogen play in this scheme?

## The rodent optic nerve model

In our studies of brain energy metabolism we have chosen the rodent optic nerve as a model of central white matter (Ransom et al., 1997). In the adult human brain white matter occupies 50% of brain volume whereas the equivalent is only about 15% in the rodent, a ratio that clearly illustrates the high degree of inter-regional connectivity (Zhang and Sejnowski, 2000; Karbowski, 2003). Thus, any metabolic disturbances within the brain are likely to affect both gray and white matter areas. The optic nerve offers the following advantage over other white matter tissue, such as the corpus callosum and the ventral column of the spinal cord, in that it is easy to remove without damaging the tissue. An additional advantage is that the stimulus-evoked response recorded from the optic nerve is a stereotypical triple peaked profile that offers a stable baseline against which the compound action potential (CAP) resulting from experimental interventions can be compared (Stys et al., 1991), whereas corpus callosum and spinal cord white matter tracts are far more prone to variability in stimulus evoked potentials due to the plane in which the section is cut and degree of injury incurred by the tissue during slicing (Baltan, 2006; Velumian et al., 2011). The optic nerve comprises myelinated axons (almost all axons are myelinated in the rodent adult) (Ransom et al., 1997), oligodendrocytes and astrocytes, and as such it is a simple model system without the complications of synapses or neuronal cell bodies. In our experimental set-up the acutely isolated optic nerve is maintained in an oxygenated chamber at 37°C and superfused with bicarbonate buffered artificial cerebrospinal fluid (aCSF) containing 10 mM glucose. Under these circumstances a stable CAP can be recorded for many hours (Brown et al., 2003b). *In vitro* experimental studies have demonstrated that both glucose and lactate (20 mM lactate is the carbon equivalent of 10 mM glucose) can fully support the CAP for extended periods of time (Brown et al., 2003b). When these experiments commenced over a decade ago the role of oligodendrocytes was ignored with axons and astrocytes of primary interest, thus only these cells shall be addressed. That glucose can support function is to be expected, and given the intimate regulatory roles that astrocytes play in supporting neural elements, significantly their role in buffering [K^+^]_o_ (Hoppe et al., 1991), we conclude that both astrocytes and axons can take up glucose, and metabolize it efficiently such as to promote the continued maintenance of the cell i.e., maintain their trans-membrane ion gradients. In the absence of sufficient glucose-derived ATP the Na pump would cease to function and the trans-membrane ion gradients would be lost, leading to a decrease and eventual loss of the CAP (Leppanen and Stys, 1997; Stys, 1998). Substituting glucose with lactate had no material impact on the CAP suggesting that lactate can be taken up and utilized by both axons and astrocytes for extended periods of time. These experiments do not, however, cover the role of substrate transfer between axons and astrocytes. For example supplying the nerve with glucose could result in astrocytes taking up glucose and converting it to lactate, which is then shuttled to the axons (this is in part what the astrocyte neuron lactate shuttle hypothesis proposes); in such a condition although supplying exogenous glucose supported axon function the axons did not take up glucose directly, but survived on astrocyte-derived lactate. To circumvent such arguments we used the compounds D-lactate and cinnemate, which block lactate transport, D-lactate as a competitive inhibitor and cinnemate as a conventional blocker. Neither compound affected the ability of glucose to support the CAP (Brown et al., 2003a), thus we feel we can safely assume that axons and astrocytes can directly take up glucose. These assumptions are supported by the presence of the glut-1 glucose transporter on astrocyte membranes (Morgello et al., 1995). The argument for lactate being used independently by both axons and astrocytes is that it is unlikely a cellular compartment would take up lactate and subsequently release it, given that the uni-directional transport of lactate and H^+^ is controlled by their respective trans-membrane ion gradients (Halestrap and Price, 1999).

The dogma of cellular compartmentalization of metabolism is that neural elements are oxidative and astrocytic elements are glycolytic, thus astrocytes produce lactate, which is consumed by neurons. There is a considerable body of evidence to support this simplistic assumption, although much of it derives from tissue culture studies, which must be viewed with caution, as a very important aspect, namely the intercommunication between cell types, is absent in these types of study. There are many studies showing that astrocytes in culture do release lactate when bathed in a medium containing glucose (Dringen and Hamprecht, 1992; Dringen et al., 1995). Similar studies of cultured neurons tend to show that neurons take up, rather than release lactate (Schurr et al., 1988, 1997a,b). Indeed lactate fuels both neuronal recovery after hypoxia (Schurr et al., 1997a), and is consumed by neurons in culture at rest, in the presence of normoglycemic glucose (Bouzier-Sore et al., 2006). In our rodent optic nerve model we investigated the trafficking of lactate between astrocytes and axons (neural elements), by investigating the role of glycogen in supporting axon conduction. It is recognized that in adult mammalian brain the glycogen resides primarily in astrocytes (Cataldo and Broadwell, 1986), a serendipitous location as it allows us to pinpoint the cellular location of glycogen-derived lactate. Our initial study localized glycogen to astrocytes, and biochemical assay revealed significant depots of glycogen in the tissue. The glycogen content was labile, increasing commensurately with elevations in bathing glucose concentration. Under conditions designed to simulate *in vivo* aglycemia, when aCSF lacking glucose was superfused, the CAP was lost, but only after a significant latency of 20 min in mouse (Brown et al., 2003a), and 30 min in rat (Wender et al., 2000). The latencies could be altered by experimentally manipulating the tissue glycogen content prior to aglycemia, such that elevated glycogen content led to increased latencies and vice versa (Brown et al., 2003a). Biochemical assay revealed that at the onset of aglycemia glycogen content fell and continued to fall until it reached its nadir, which coincided with the loss of the CAP (Brown et al., 2003a) (Wender et al., 2000). These results showed that tissue glycogen was metabolized and supported axon conduction in the absence of exogenously applied glucose, but that once the glycogen was exhausted the CAP failed. The nature of the support afforded by glycogen was investigated, based on assumptions drawn from previously published data, namely that astrocytes produce lactate and neural elements consume lactate. For this to be the case several conditions must be met, which can be tested experimentally. First, the astrocytes and axons must express the means to transport lactate i.e., monocarboxylate transporters (MCTs). There are four isoforms of this transporter (Halestrap, 2012), but we focused on the two isoforms most likely to be involved, MCT1, which is expressed in tissue that releases lactate, and MCT2 that is expressed on tissue that takes up lactate. Immunohistochemical data using specific antibodies that recognize MCT1 and MCT2 in combination with cell specific markers, namely GFAP for astrocytes and neurofilament for axons, showed expression patterns supportive of astrocyte to axon lactate transport, namely axonal MCT2 expression and astrocytic MCT1 expression. In addition the expression pattern of the enzyme lactate dehydrogenase demonstrated neuronal expression of the LDH1 isoform, whereas astrocytes expressed both LDH1 and LDH5 isoform indicating that both cell types are capable of inter-converting lactate and pyruvate (Tekkok et al., 2005).

These experiments convincingly demonstrate glycogen-derived lactate transport from astrocytes to axons under conditions of aglycemia. What is of more interest is to determine the role of glycogen under more physiological conditions, such as hypoglycaemia, which is a common side effect experienced by type 1 diabetic patients who use exogenous insulin to manage their symptoms (Bittar et al., 1996), and under increased tissue energy demand. Mouse optic nerves superperfused with 10 mM glucose can sustain conduction for many hours. Switching to 2 mM glucose aCSF had no effect on the CAP (Frier and Fisher, 2007). However, adding cinnemate, which blocks lactate transport at the MCT causes a decrease in CAP area (Brown et al., 2003a). In a separate experiment superfusing nerves with 2 mM glucose after previously depleting glycogen with 20 min of 0 glucose superfusion caused the CAP to fall (Brown et al., 2003a). Our conclusion from these experiments is that 2 mM glucose is insufficient to fully support the CAP and that glycogen is metabolized in the astrocyte to lactate, which is then shuttled to the axon to maintain axon conduction. Interrupting lactate transport or decreasing glycogen content results in CAP failure. The mouse optic nerve is able to sustain firing when stimulated at frequencies of up to 50 Hz i.e., the CAP remains above baseline during sustained stimulation. At 100 Hz the CAP area initially increased above baseline due to the CAP spreading out (Brown et al., 2003a) as a result of increased extracellular K^+^ accumulation (Brown et al., 2003a), followed by a slow decrease below baseline after about 5 min. Addition of isofagomine, an inhibitor of glycogen phosphorylase (Ransom et al., 1985), or D-lactate, or cinnemate (Brown et al., 2005), caused a decrease in the CAP, indicative of glycogen metabolism sustaining the CAP during periods of increased energy demand. Thus, the increased tissue energy demand invoked by imposing high frequency stimulus on the nerve, is not met by ambient glucose concentrations, and glycogen derived lactate provides supplementary energy substrate. An alternative view has been proposed whereby astrocytes metabolize glycogen thus maintaining high levels of glucose-6-phosphate, diverting glucose for neuronal oxidation (Dienel, 2012).

Thus, in the rodent optic nerve any lactate present in the extracellular space is likely to be due to glycogen metabolism under conditions of increased metabolic need by axons. However, we are interested in whether glucose contributes to the putative extracellular lactate pool. That astrocytes release lactate, which is taken up by neural elements has a firm foundation backed up by experimental evidence as described above. However, in the mid 1990's the astrocyte neuron lactate shuttle hypothesis (ANLSH) proposed that a significant proportion of glucose delivered to the brain was shuttled into astrocytes where it was converted to lactate and subsequently released into the extracellular space and taken up by neurons (Dienel and Cruz, 2014). However, this scheme proposed an “on demand” aspect, such that the initiating factor for the sequence was release of glutamate into synapses, with the glutamate subsequently taken up by astrocytes, which provoked glucose uptake. As such the scheme would appear to propose that under resting conditions there was little lactate trafficking between astrocytes and neurons, and that this was only initiated by metabolic need by neurons, and under conditions of increased energy demand both glucose and glycogen are proposed to be sources of lactate (Pellerin and Magistretti, 1994). Recent data has suggested that the brain does not use lactate as an obligate substrate to the extent that the brain can release lactate into the systemic circulation, and lactate is only used when present in high non-physiological concentration (Magistretti et al., 1999).

## Lactate biosensors

**Figure 1:**
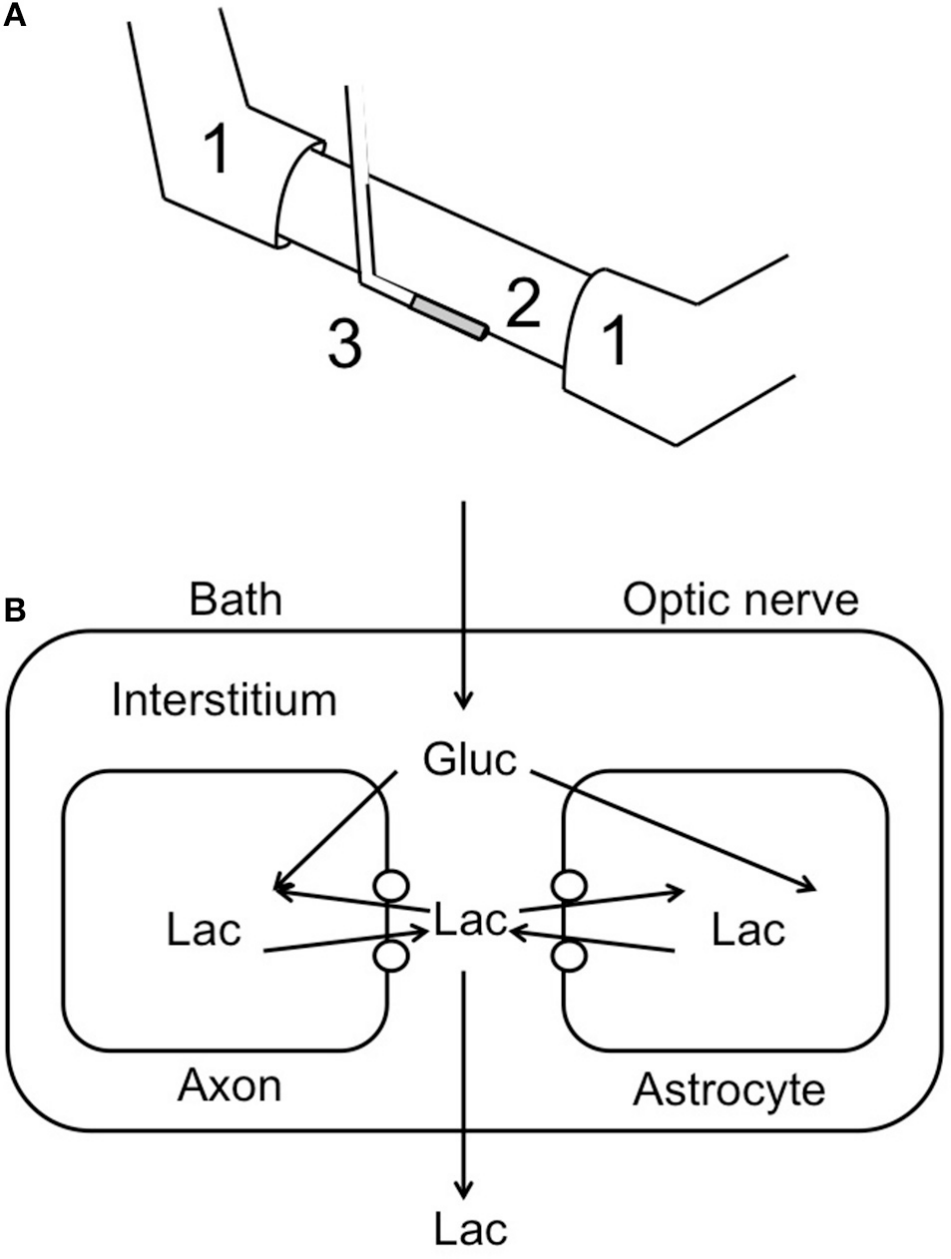
**Recording lactate release from optic nerve**. **(A)** Schematic illustrating the use of a lactate biosensor (3) to record lactate release from optic nerve (2). The CAP is recorded via the suction electrodes (1). **(B)** A model of possible lactate movements in the continually superfused optic nerve. The open circles indicate monocarboxylate transporters; Gluc, glucose; Lac, lactate.

**Figure 2:**
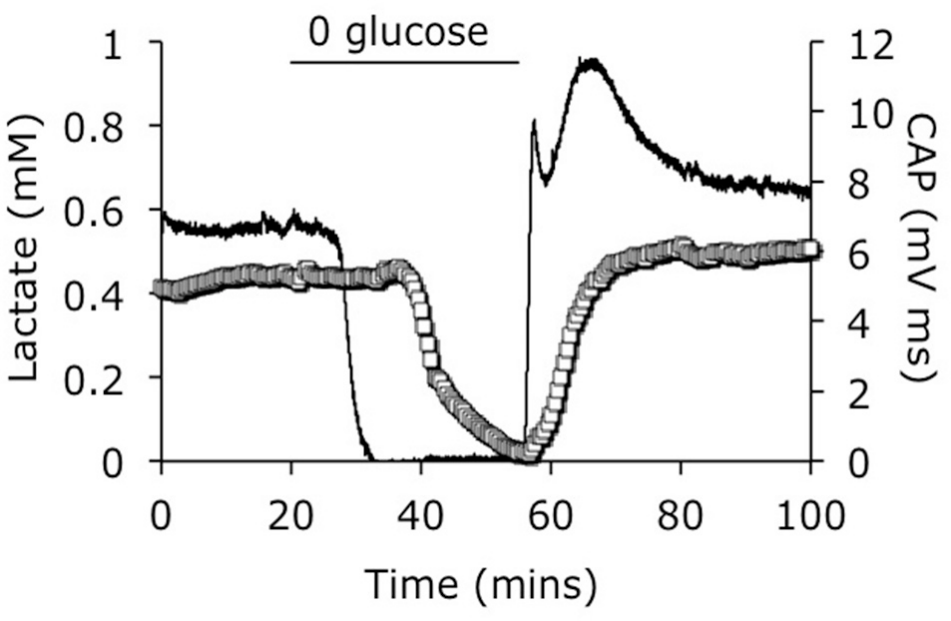
**Lactate concentration in response to aglycemia**. A baseline period of superfusion with aCSF containing 10 mM glucose produced a stable CAP area and a robust release of lactate from the tissue. Introduction of aglycaemia caused a rapid fall in lactate to zero, prior to the fall in the CAP area. On reperfusion of glucose containing aCSF the CAP rapidly recovered, but the lactate level rapidly overshot before returning to the baseline levels. The CAP (open squares) is represented on the left y-axis and the lactate signal (line) is represented on the right y-axes. Adapted from Figure 4 Yang et al. (2014).

The development of lactate biosensors offered an ideal opportunity to investigate the dynamics of extracellular lactate. The rodent optic nerve is an ideal preparation to use as the biosensor can be placed alongside the nerve, i.e., just outside the meninges, without damaging the tissue (Yang et al., 2014). It is worthwhile examining this configuration in detail as illustrated in Figure 1. In such a configuration the sensor does not record lactate in the interstitium but rather at the periphery of the nerve. Thus, any lactate recorded has been released by the nerve and cannot be used as a substrate i.e., it can be considered surplus to current energy requirements. Given the superfusion system, there is a constant flow of aCSF over the sensor, thus the sensor would record any lactate released by the tissue into the aCSF. What this means is that a stable reading of lactate implies there is a constant efflux of lactate from the nerve, a decreased level means a decreased release and an increase means an increased release. Compare this to a static enclosed system, where an acute release of lactate would be recorded as a continuously elevated level. With this in mind it is timely to discuss the implications of recent recordings using lactate biosensors in mouse optic nerve. It is immediately apparent that in nerves perfused with control concentrations of glucose the lactate concentration was about 500 μM (the concentration may not be an accurate reflection of the interstitial [lactate] due to continuous superfusion and incomplete contact of the sensor with the nerve etc.). That such a large concentration is recorded may be viewed as surprising, but is in agreement with *in vivo* data using dialysis techniques that have recorded equivalent concentrations (Hu and Wilson, 1997). However, the *in vivo* situation is different as the ECS is a stable compartment, and dependent upon the rate of release or uptake of lactate, may not reflect a vibrant flux of lactate, but rather a stationary lactate pool. Alternatively, the conditions present in these experiments in which aCSF lacking lactate superfuses the tissue may exaggerate any true release of lactate from the tissue due to the extracellular pool lacking lactate. In addition it has recently been shown that extracellular lactate inhibits astrocytic glycolysis (Sotelo-Hitschfeld et al., 2012). Another intriguing possibility, although we present no evidence to support it, is that white matter is a lactate producer and gray matter is a lactate consumer. Such a high level of recorded lactate release warrants comment, and strongly suggests that the tissue creates and exports lactate to the interstitium irrespective of metabolic need. This contradicts the ANLSH, where lactate is exported by astrocytes on demand by neurons (Magistretti et al., 1999). The compartmentalization of lactate production has not been elucidated in this system, but it is highly likely that the glial (astrocytic) component produces most of the lactate, and thus exports lactate as a continuous process. That the lactate is released and unutilized is initially surprising, but appears logical on closer inspection. An axon in an *in vivo* situation that suddenly increases its energy demand would be fed by an increase in localized blood flow that is initially via Ca^2+^ waves in astrocytes, and as such is not an instantaneous process taking between tens of seconds and minutes. In the latency between this occurring the axons may be bereft of instantly available glucose if relying in ambient glucose. However, an additional pool of extracellular lactate may be an ideal reserve from which axons feed prior to hyperemia. Introduction of aglycemia to mouse optic nerves led to a rapid fall in lactate to zero after about 10 min. However, this preceded the fall in the CAP suggesting that lactate is taken up by the axons during aglycemia, but that once it has been used there is no energy substrate for the axons to use, and hence CAP failure ensues. On reperfusion of glucose the lactate rapidly increases, overshoots, then returns to baseline (Figure 2). The mechanism behind this overshoot is unknown but may be in part due to the aglycemia-induced elevated extracellular K^+^ concentration (Brown et al., 2001), which is supported by recent data showing K^+^ stimulated glycogen degradation in astrocytes mediated by soluble adenyl cyclase (Choi et al., 2012), and astrocyte specific bicarbonate-sensitive NBCe1-mediated stimulation of glycolysis (Bittner et al., 2011; Ruminot et al., 2011).

In conclusion, glycogen supports axon conduction in both peripheral and central axons. The glycogen is located in the glial compartment in each tissue, and is metabolized to the transportable conduit lactate, which is shuttled to the axons to support conduction. Such metabolic cell-to-cell communication relies on signaling between the cells such that reliable information is relayed to the glial cells concerning the current metabolic requirements of the neural elements. Our knowledge of the mechanism(s) of this communication is incomplete, but the use of reduced models of brain tissue in combination with emerging technologies will surely advance our understanding of the fascinating and complex interaction between neurons and glia.

### Conflict of interest statement

The authors declare that the research was conducted in the absence of any commercial or financial relationships that could be construed as a potential conflict of interest.
